# Circulating Free DNA as Biomarker and Source for Mutation Detection in Metastatic Colorectal Cancer

**DOI:** 10.1371/journal.pone.0108247

**Published:** 2015-04-13

**Authors:** Karen Lise Garm Spindler, Niels Pallisgaard, Rikke Fredslund Andersen, Ivan Brandslund, Anders Jakobsen

**Affiliations:** 1 Department of Oncology, Vejle Hospital, Vejle, Denmark; 2 Department of Biochemistry, Vejle Hospital, Vejle, Denmark; Sun Yat-sen University Cancer Center, CHINA

## Abstract

**Background:**

Circulating cell-free DNA (cfDNA) in plasma has shown potential as biomarker in various cancers and could become an importance source for tumour mutation detection. The objectives of our study were to establish a normal range of cfDNA in a cohort of healthy individuals and to compare this with four cohorts of metastatic colorectal cancer (mCRC) patients. We also investigated the prognostic value of cfDNA and analysed the tumour-specific *KRAS* mutations in the plasma.

**Methods:**

The study was a prospective biomarker evaluation in four consecutive Phase II trials, including 229 patients with chemotherapy refractory mCRC and 100 healthy individuals. Plasma was obtained from an EDTA blood-sample, and the total number of DNA alleles and *KRAS* mutated alleles were assessed using an in-house ARMS-qPCR as previously described.

**Results:**

Median cfDNA levels were higher in mCRC compared to controls (p <0.0001). ROC analysis revealed an AUC of 0.9486 (p<0.00001). Data showed impaired OS with increasing levels of baseline cfDNA both when categorising patients by quartiles of cfDNA and into low or high cfDNA groups based on the upper normal range of the control group (Median OS 10.2 (8.3–11.7) and 5.2 (4.6–5.9) months, respectively, HR 1.78, p = 0.0006). Multivariate analysis confirmed an independent prognostic value of cfDNA (HR 1.5 (95% CI 1.3–1.7) for each increase in the cfDNA quartile). The overall concordance of *KRAS* mutations in plasma and tissue was high (85%).

**Conclusions:**

These data confirm the prognostic value of cfDNA measurement in plasma and utility for mutation detection with the method presented.

## Introduction

Metastatic colorectal cancer (mCRC) holds a poor prognosis and despite recent improvements resistance to therapy is still a major challenge. The search for better selection criteria for therapy along with new potentially effective treatment regimens for chemotherapy-resistant disease has drawn considerable attention during the last decade. These include development of new agents, identification of genetic alterations responsible for resistance and search for biomarkers for guidance during therapy.

The presence of circulating cell-free DNA (cfDNA) in the blood was reported more than 60 years ago [1]. It is actively released from normal and deceased cells, apoptosing and necrotising processes, as well as from complex interactions between tumour and adjacent non-tumour cells[2–5]. Cell-free DNA can be detected in serum, plasma and other body fluids[6], but the mechanisms of release into the blood stream and the origin of the DNA are far from fully understood. Further clarification is required to make a reliable distinction between malignant increases and non-cancerous variations in cfDNA.

Studies have suggested that the level of cfDNA is increased in both cancer patients[7–8] and in various non-malignant pathological conditions compared to healthy individuals. However, a recent meta-analysis demonstrated inconsistent results[9]. Establishing a normal range is therefore a prerequisite for further investigation of the potential role of cfDNA as a diagnostic marker, as well as of its utility in the early detection of recurrences.

Cell-free DNA has also been considered a potential prognostic marker for outcome in various cancers[10]. Recently, we reported that cfDNA held prognostic value in patients with mCRC[11–13]. A high number of cfDNA alleles in the plasma consistently correlated with a poor overall survival (OS) in our patients treated with thirdline chemotherapy for mCRC, whereas patients with a low plasma concentration of cfDNA had a longer median OS. Verification of these results in larger cohorts is highly relevant in establishing the clinical potential of cfDNA.

In addition to its potential as a diagnostic tool and prognostic marker, cfDNA is also a valuable source for detecting tumour-specific mutations in the peripheral circulation of cancer patients[14–16]. In mCRC, there is a high frequency of *KRAS* mutations, which are responsible for resistance to the widely used monoclonal antibodies targeting the EGFR[17]. Molecular analysis of genomic alterations are normally performed on archival tumour tissue, but there have been concerns that this approach does not sufficiently reflect the disease biology at the time of initiation of targeted EGFR therapy, which is often several years from the primary diagnosis and/or surgery. Moreover, repeated biopsies are not feasible for practical and ethical reasons. Hence, the use of cfDNA for detecting these tumour-specific mutations may be an attractive addition for better patient selection for targeted therapies in the future.

The methods used for DNA quantification have varied over time, ranging from simple qPCR methods to complex BEAMing technologies and deep next generation sequencing[18,5]. The sensitivity and specificity of analysis have improved many folds since the initial studies, but the uses of different sampling materials and methods for cfDNA quantification, in addition to inconsistent reporting, have complicated a valid comparison of the results from different studies. Recent advances in technological methods have enabled us to develop a highly sensitive qPCR method for quantifying cfDNA in plasma samples, which is also feasible in the laboratory. This has allowed us to investigate the biomarker potential of cfDNA in a large cohort of cancer patients and healthy controls.

The objectives of the present study were to establish a normal range of cfDNA in a cohort of healthy individuals and to compare this cfDNA range with those from four different cohorts of patients treated for mCRC. In addition, we aimed to validate the prognostic value of the pre-treatment levels of cfDNA and analyse the tumour-specific *KRAS* mutations in the plasma.

## Materials and Methods

### Study design

A total of 100 healthy individuals and 229 mCRC patients were investigated. The study was performed as a prospective biomarker investigation in four consecutive Phase II and biomarker studies: the Cetuximab study[11,20], the TIRASMUS (ClinicalTrials.gov identifier; NCT00827684, [12], PG (ClinicalTrials.gov identifier; NCT01109615 [13] and GemCap (ClinicalTrials.gov identifier; NCT01472770, [32] trials conducted at the Department of Oncology, Vejle Hospital, Denmark, from April 2005 to November 2012. All four phase II studies included patients with chemotherapy refractory disease and biomarker collection was prospectively conducted.

The primary endpoint was correlation of cfDNA to OS, secondary progression free survival (PFS) and evaluation of the difference between healthy controls and mCRC patients, in addition to the correlation between tumour KRAS (tKRAS) and plasma KRAS (pKRAS) detection. Data are presented according to REMARK guidelines.

### Patients

The inclusion criteria in the trials were similar: histopathologically verified stage IV mCRC, treatment failure after exposure to flouropyrimidine, oxaliplatin and irinotecan, indication for third or fourth line treatment, ECOG performance status (PS) 0 to 2, and adequate organ function. *KRAS* and *BRAF* status determined inclusion in the TIRASMUS and PG trials, but not in the Cetuximab or GemCap studies.

RECIST version 1.1 and NCI-CTCAE version 4.0 were used to examine the endpoints in the GemCap and PG studies, whereas in previous trials, RECIST version 1.0 and NCI-CTCAE version 3.0 were used.

All patients provided signed informed consent before study entry and the relevant regulatory and ethics committees (Danish Medicines Agency and The Regional Scientific Ethical Committee for Southern Denmark) approved the studies prior to commencement. The clinical trials were conducted in accordance with the Good Clinical Practice guidelines as issued by the International Conference on Harmonization and the Declaration of Helsinki.

### Treatment

#### The Cetuximab Study

Treatment comprised irinotecan (350 mg/m^2^) every three weeks, with weekly 250 mg/m^2^ of cetuximab (initial loading dose was 400 mg/m^2^), until progression or unacceptable toxicity. Response evaluation was performed every three cycles of therapy.

#### TIRASMUS

Patients received treatment with the mTOR inhibitor temsirolimus 25 mg every week until progression. Thereafter, patients were treated with combination therapy comprising biweekly irinotecan (180 mg/m^2^) and weekly temsirolimus until progression or unacceptable toxicity. Response evaluation was performed every 6 weeks.

#### PG

Patients received pemetrexed (initially 500 mg/m^2^ q3w) + gemcitabine (1250 mg/ m^2^, days 1 and 8) until progression or unacceptable toxicity. Response evaluation was performed every three cycles of therapy.

#### GemCap

Patients received capecitabine (2000 mg/m^2^, day 1–7, q2w) combined with gemcitabine (1000 mg/m^2^, day 1) until progression or unacceptable toxicity, and response was evaluated every 12 weeks.

### Healthy control group

The healthy individual cohort was selected from the Diabetes Biobank Vejle (approved by the Danish Data Protection Agency (journal no. 2006-53-1385) and the Danish Scientific Committee (project ID S-20080097)).

Individuals were selected based on age groups, and The National Danish Patient Register was accessed for ICD-10 codes to ensure a lack of significant co-morbidity (ICD-10 C, D, I and M codes). Informed consent was obtained from each individual for the use of blood samples, and the study approved by the Regional Scientific Ethics Committee for Southern Denmark for this additional marker analysis.

### Sampling for translational research studies

Collection of primary tumour tissue and blood samples for translational research is a standard procedure in trials conducted in our department. After informed consent, pre-treatment blood samples were drawn prior to the first cycle of therapy. The methods for quantification of cfDNA and mutated *KRAS* alleles in the plasma have been previously described[11], and information of primers and probes are available online. Plasma was obtained from blood samples collected in EDTA-tubes and centrifuged at 2000 *g* for 10 min within 2 hours of collection, before being stored at -80°C until use. All samples were analysed, blinded to the study endpoints.

### DNA purification

DNA was purified from 1 ml of plasma using a QIAsymphony virus/bacteria midi-kit on a QIAsymphony robot (Qiagen), according to the manufacturer’s instructions. DNA was eluted in 110 ul. AVE buffer which was supplied with the kit.

### Cell-free DNA quantification

To determine the level of cfDNA, the amount of the peptidylprolyl isomerase A (cyclophilin A) gene (gCYC, HUGO gene abbreviation PPIA) was measured by an in-house qPCR assay as described in [11]. Assays were run in dublicates or triplicates and 5 ul of DNA was used in each 25 ul PCR reaction. For each PCR a pool of genomic DNA was included as positive control and water as negative control. The CV of the gCYC assay based on the positive control pool was determined to be 19%. Water controls were always negative.

Primers and probes for the in-house gCYC were designed using the OLIGO 7 software (Molecular Biology Insights Inc, Cascade, CO) (available online, sequence accession number NG_029697.1). The forward primer is located in intron 1–2, the probe in exon 2 and the reverse primer in intron 2–3 (Forward ACATGGGTACTAAGCAACAAAATAAG, Reverse CACAATTGGAACATCTTTGTTAAAC, Probe Fam-TTGCAGACAAGGTCCCAAAGACAGCA-Tamra). BLAST search was performed and no unspecific targets were predicted. Analysis of qPCR data was done using SDS software ver. 2.2.2 and Cq values were determined using automatic baseline setting and fixed threshold at 0.2. Quantification of cfDNA was done by calculating the copy number of *gCYC* alleles as 10^y-intercept(gCYC)—mean Cq(gCYC)/slope(gCYC)^ and normalizing this to the plasma volume.

### KRAS mutation detection and quantification

*KRAS* analysis of archival tumour tissue from FFPE was performed in the Cetuximab study with the FDA-approved *KRAS* DxS kit (Qiagen), as previously reported. Tumour samples from the TIRASMUS, PG and GemCap trials were analysed with a validated in-house assay. The in-house assays are based on the Amplification Refractory Mutation System-Quantitative PCR (ARMS-qPCR) methodology and detects 6 mutations in *KRAS* codon 12 (Gly12Ala, Gly12Arg, Gly12Asp, Gly12Cys, Gly12Ser, and Gly12Val) and one mutation in codon 13 (Gly13Asp). Primers and probes for the in-house *KRAS* assays, as well as *KRAS* mutation control PCR fragments, were designed using the OLIGO 7 software (Molecular Biology Insights Inc, Cascade, CO) (available online, sequence accession number NW_001838052.1). The assay is located in exon 2 of the KRAS gene. Analysis was performed as described above. A comparison of the two methods for mutation detection in tumour tissue has been published previously and showed complete agreement[19] and the standard curves are available as online material[11]. The detection sensitivity varied between 0.03%–0.001% depending on the type of mutation detected, 12asp (1/200000, 0.0005%), 12Cys (1/200000, 0.0005%), 12ser 1/7000, 0.0143%), 13asp 1/3000, 0.0333%), 12ala (1/100000, 0.0010%), 12val (1/200000, 0.0005%), 12arg (1/200000, 0.0005%), respectively). A mixture of mutation positive control fragments and genomic donor DNA was included in each PCR run and water as negative control. A sample of pure genomic donor DNA was included to determine the specificity of the assays (data presented in[11]). CV for each KRAS mutation assay and for the gCYC assay was determined based on data from PCR analyses over a time period of 20 months and were between 18% and 32%. Water controls were always negative. Quantification of *KRAS* was done by calculating the copy number of mutated *KRAS* alleles as 10^y-intercept(KRAS)—mean Cq(KRAS)/slope(KRAS)^ and normalizing this to the plasma volume.

### Statistical analysis

A descriptive comparison of cfDNA levels was performed with two-sided t-test and Wilcoxon rank sum test. The upper normal limit, as defined by the mean +2SD in the control group, was used for the explorative cut-off value for survival analysis. The Kaplan-Meier method was applied to estimate PFS and OS. A multivariate Cox regression analysis was performed to examine whether the different variables were associated with reduced survival, including cfDNA levels and testing for baseline characteristics (age, gender and PS) with potentially influence (known prognostic factors or significant or borderline parameters e.g. p<0.02) on survival. A receiver operating curve (ROC) analysis was employed to describe the performance of cfDNA and pKRAS. P-values referred to two-tailed tests and were considered significant when p ≤ 0.05. Statistical analyses were carried out using the NCSS statistical software 2007 v.07.1.5 (NCSS Statistical Software, Utah 84037, USA, www.ncss.com).

## Results

### Patient characteristics

Disease and pre-treatment patient characteristics are presented in **Table 1**. The median age in the total cohort of patients was 63 years (range 35–82) and the majority were male, mostly in the good performance status. There were no significant differences in age, gender or performance status between the 5 cohorts.

**Table 1:** Pre-treatment characteristics and cell-free DNA levels in all cohorts.

Characteristic		Number	(%)	cfDNA no alleles/ml	p-value
		Total (#)		Median (Range)	
**Age** *					
	≤ 63 years	123 (119)	54	14900 (1000–2259600)	
	> 63 years	106 (104)	46	21000 (800–4618400)	0.39
**Gender** *					
	Female	99 (97)	43	25000 (1000–4618400)	
	Male	130 (126)	57	15400 (800–1203100)	0.10
**ECOG performance status** *					
	0	109 (107)	48	13100 (800–1020400)	0.04 (0vs1)
	1	109 (107)	43	22500 (2200–4618400)	0.02 (0vs2)
	2	19 (19)	8	29600 (5365–627056)	0.12 (1vs2)
**Study cohort**					
Cancer patients		229 (223)	100		
	Cetuximab	108 (106)	47	23100 (2000–4618400)	
	TIRASMUS	32 (30)	14	11000 (800–1203100)	
	PG	40 (40)	18	19800 (2200–2259700)	
	GemCap	49 (47)	21	13200 (1000–549500)	
Healthy controls		100 (99**)		2400 (800–14200)	<0.0001***

Data have been rounded to nearest 100.

# total number with available samples,

* only cancer patients

**One sample excluded because of visible hemolysis

*** All p-values for differences between each cancer cohort and the control group

The healthy individual cohort included 10 males and 10 females aged 25–44 years, 20 males and 20 females aged 45–59 and 60–75.

The majority of the patients included in the trials had available blood samples. Thus, a total of 229 patients were included from the four trials, and 223 had available tumour tissue and 211 a matching baseline plasma sample. The missing results were due to poor quality tumour DNA or due to inadequate amount of plasma in the samples.

### Cell-free DNA and pre-treatment characteristics

The median cfDNA levels in the individual cohorts are given in **Table 1**. Cell-free DNA levels were analysed for correlation to disease and pre-treatment characteristics. There were no significant differences in median cfDNA levels according to age or gender, but significantly higher cfDNA concentrations with increasing PS (**Table 1**).

### Cell-free DNA in cancer patients compared to healthy individuals

**Fig 1:**
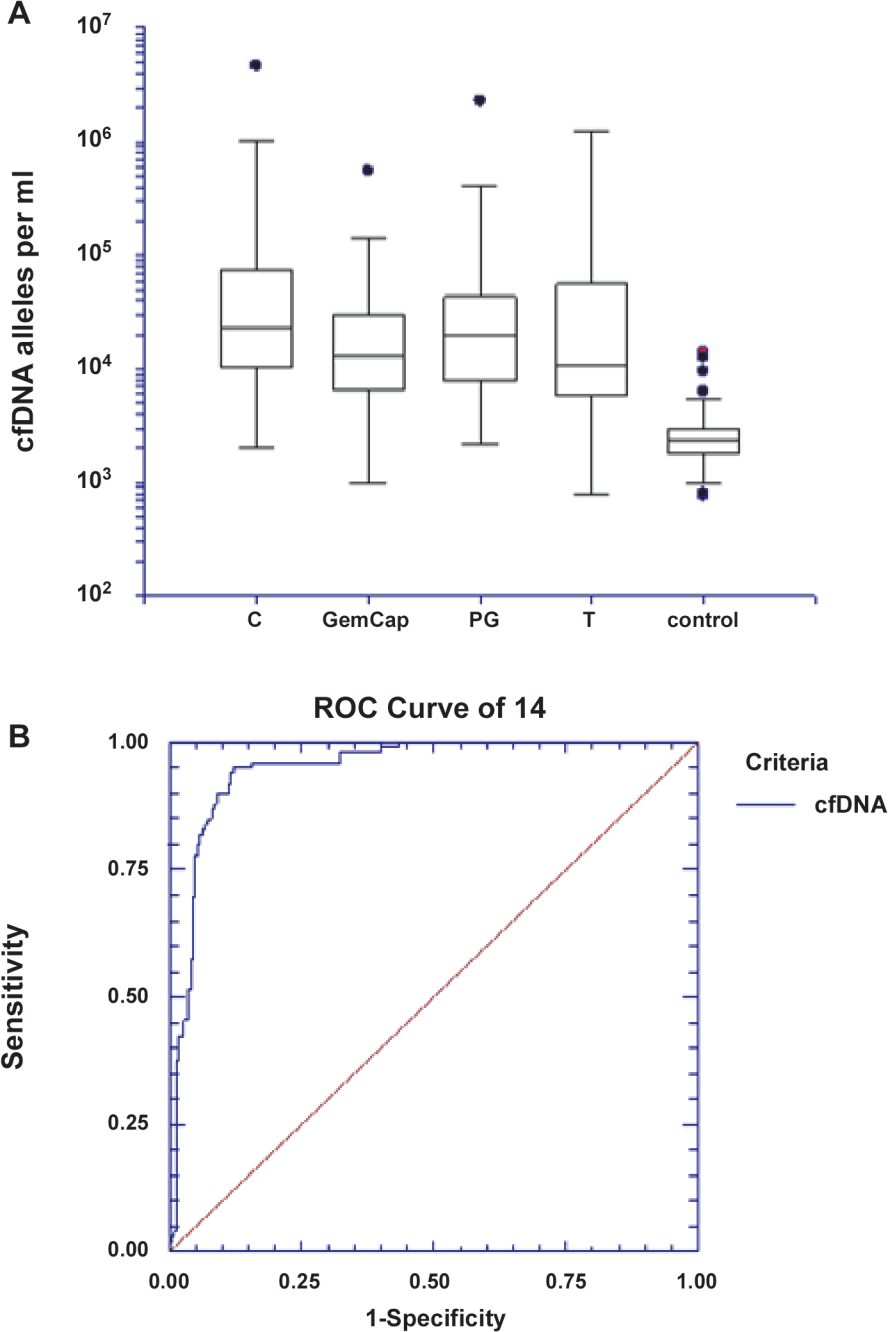
A and B. Cell free DNA concentrations in colorectal cancer cohorts and healthy controls. **A** depicts Box and whisker plots with 25%, 50%, 75% percentiles, upper and lower adjacent values and outliers (dots). Horizontally the four clinical trial cohorts and control group, vertically the cfDNA concentration on a logarithmic scale. C cetuximab study, GemCap GemCap cohort, PG PG study cohort, T TIRASMUS study cohort, Controls healthy control group. **B** Shows a receiver operating curve (ROC) estimating the performance of cfDNA to discriminate between colorectal cancer patients and controls. The AUC was 0.9486, (95% CI 0.9182–0.9679, p<0.00001).

The mean and median concentrations of cfDNA in the control group were 2800 and 2400 alleles per ml of plasma, respectively, within a range of 800 to 14000 alleles per ml of plasma. The median level of cfDNA in the entire cohort of cancer patients was 17900 alleles per ml of plasma (range 800–4618400). **Fig 1A** illustrates the differences in cfDNA concentrations in the plasma of healthy controls and the baseline samples from the different cohorts of cancer patients. Data are presented as a box plot of values on a logarithmic scale, and the levels in cancer patients were markedly higher than in the healthy control group. The levels of cfDNA were similar between the cohorts of cancer patients. The differences in cfDNA were highly significant between the control group and all individual groups of cancer patients (all p values were <0.0001). The power to discriminate between healthy individuals and cancer patients was tested by estimating the ROC and area under the curve (AUC) as demonstrated in **Fig 1B**. The AUC was 0.9486 (95% CI 0.9182–0.9679, p<0.00001).

### The prognostic value of cfDNA in cancer patients

**Fig 2:**
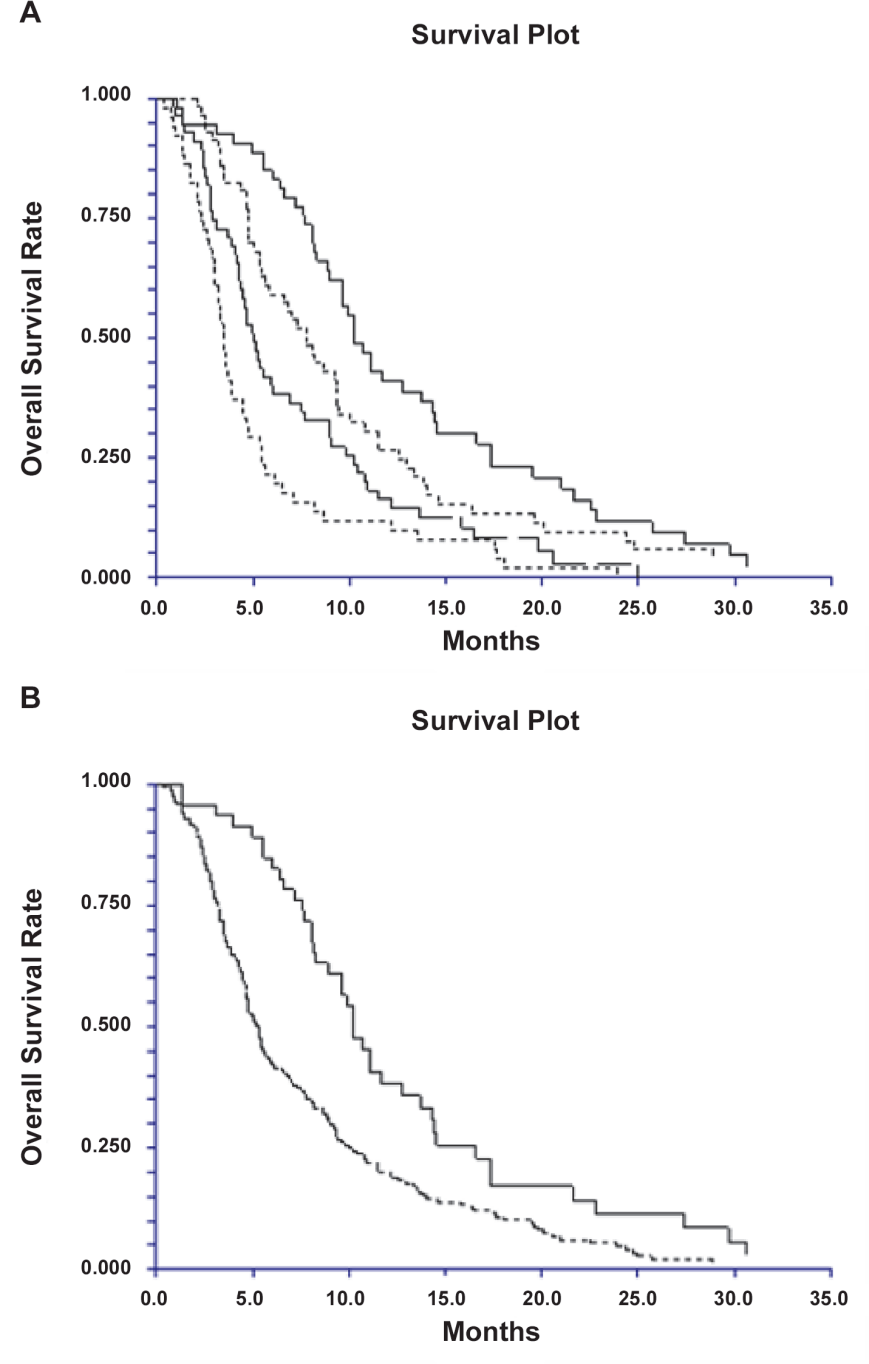
Kaplan Major Plots of OS probabilities. A. Patients are grouped by quartiles of cfDNA (from the right) lowest, second lowest, second highest and highest quartile of cfDNA. The median OS according to cfDNA quartiles were; 10.2 months (95% CI 8.9–12.8), 7.8 months (5.7–9.3), 5.0 months (4.3–6.0) 3.5 months (3.0–3.9), respectively. B. Patients are grouped by the upper normal range as defined by mean cfDNA+2SD in the control group (7100 alleles per ml.) Low risk group (full line), Median OS, 10.2 months (8.3–11.7). High risk group (dotted line) Median OS, 5.2 months (4.6–5.9). HR 1.78, p = 0.0006.

Survival analysis according to cfDNA levels have are presented separately for the individual cancer groups with the primary trial results [11–13]. A pooled analysis of all patients confirmed a shorter overall survival with increasing levels of baseline cfDNA. **Fig 2A** illustrates the Kaplan-Meier curves of OS in the patients divided into the four groups according to quartiles of cfDNA levels. The OS of the patients categorised into low or high cfDNA concentration groups based on the upper normal range of the control group (defined as mean cfDNA +2SD = 7100 alleles per ml) is shown in **Fig 2B**. Cox regression multivariate analysis including age (>/< 63 years), gender, PS and cfDNA quartiles (treated as a continuous variable) confirmed an independent prognostic value of cfDNA in the entire cohort. As a continuous variable, there was an OS Hazard Ratio of 1.5 (95% CI 1.3–1.7) for each increase in the cfDNA level quartile (**Table 2**).

**Table 2:** Multivariate analysis of overall survival.

Variables		OS	
		Risk Ratio	*p*-value
		(95% CI)	
Plasma *cfDNA* quartiles**			
		1.5	<0.0001
		(1.3–1.7)	
PS *, **			
		1.5	
		(1.2–1.8)	0.0007
Age			
	< median^†^	1.0	
	> median	(0.7–1.3)	0.7782
Gender			
	Female^†^	1.2	
	Male	(0.9–1.5)	0.2862

^†^ Reference group

* PS = Performance Status (ECOG)

** Entered as a continuos variable

The risk ratio refers to moving from the reference group to the other group or changing one step in parameters entered as continuous variables.

### Detection of KRAS mutations in tumour and plasma samples

A total of 211 patients had both tumour and plasma samples available for the reliable detection of *KRAS* mutations. Plasma samples with a low number of cfDNA alleles were not excluded from the analysis. Tumour-specific *KRAS* mutations were detected in a total of 119 plasma samples. The overall concordance between mutation status in the tumour and plasma was high: (180/211 = 85%) and 112 of the 140 tumour samples with mutations demonstrated *KRAS* mutations in the plasma samples too, whereas only three cases were observed where the patients had a detectable mutation in the plasma, but not in the primary tumour.

### Cell-free DNA and correlation with tumour-specific KRAS mutations

**Fig 3:**
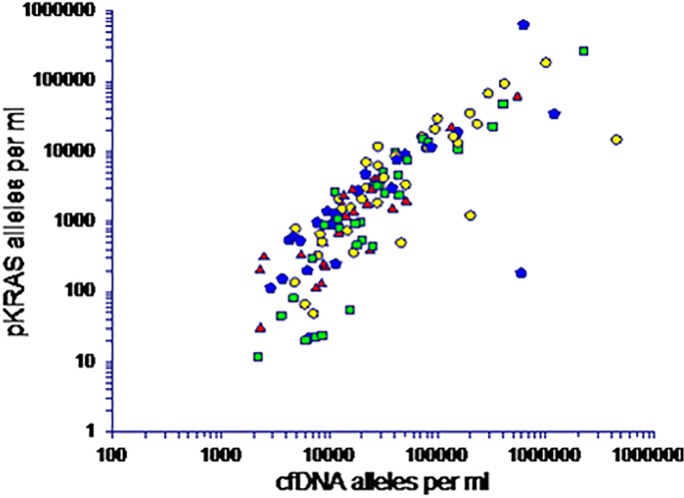
Scatter plot of correlation between cfDNA and pKRAS concentrations. This plot illustrates the strong correlation between the total number of DNA alleles and the number of mutated alleles in the patients with KRAS mutations detected. The spearman rank correlation was calculated to 0.86 and r^2^ = 0.97 (*p* < 0.0000). The different symbols represent the individual cohorts of cancer patients. (Square PG correlation = 0.9, circle cetuximab correlation = 0.88, triangle GemCap correlation = 86, pentagon TIRASMUS correlation = 0.67).

The correlation between the number of total cell free DNA alleles and the number of mutated KRAS alleles in the KRAS mutant patients was analysed by spearman rank correlation. Because of the wide range of quantitative levels a logarithmic scale was used. As shown from Fig 3, which depicts the cell free DNA vertically and the number of mutated KRAS alleles horizontally these were strongly correlated **Fig 3** (r^2^ = 0.97, Spearman rank = 0.86, p<0.000). This confirms the preliminary data from the cetuximab study [11), and indicates that a significant fraction of the cfDNA origin from tumor DNA (as represented by the DNA with tumor specific KRAS mutations)

## Discussion

Increased focus on translational research and recent technological advances have given new insight into the clinical importance of cfDNA in cancer and its potential for detecting tumour-specific mutations in peripheral circulation.

As a measure of the total amount of cfDNA, this study investigated the number of alleles of the cyclophilin gene, and confirmed a higher level in mCRC patients than in healthy controls. A search for similar studies reveals inconsistent results; however, differences in investigational methodologies make direct comparisons difficult. In general, there are variations in the method applied for DNA purification, the reference gene measured, and the cohorts of the investigated individuals. Nevertheless, a few studies in CRC patients do support our data [21–24]. A very recent study explored the multipurpose utility of circulating plasma DNA in patients with advanced cancers using a different methodological approach, showing generally higher levels of cfDNA in CRC, breast, prostate and other cancers than in healthy controls [8]. However, the sample size of that study did not allow for the establishment of a normal range (n = 20), but its results do support the present data. A recent observation in locally advanced rectal cancer, despite the use of a different method for measuring cfDNA, also revealed a significant difference in cfDNA levels between controls and early stage rectal cancer[25]. The ability to discriminate cfDNA levels between our colorectal cancer patients and healthy controls was excellent, supporting the need for further studies of cfDNA in earlier tumour stages and precancerous lesions. Furthermore, the variation of cfDNAin individuals with non-cancerous co-morbidity needs to be further elucidated, to account for potential bias. The present study also underlines the important biological role of cfDNA in this disease. The elevated concentration of cfDNA in cancer patients and correlation with a number of mutated alleles also indicate that the cfDNA is largely tumour derived, although the origin still remains undefined.

There is a need for additional tools for better selection of patients for therapy in heavily pre-treated mCRC. In four consecutively conducted clinical phase II studies we have confirmed a uniform level of cfDNA and a clear prognostic value.

A recently published study from our group has illustrated that the cfDNA is not merely a measure of the tumor burden [26]. Instead, we suggest that total cfDNA is more likely to reflect both tumor burden and biological mechanisms and is therefore a more complex picture of the disease at a certain stage. The similarity of baseline levels between the four phase II studies we observed illustrates the homogeneity of the patients’ cohorts. Patients were all selected based on true chemotherapy refractory status rather than lines of therapy, which can vary based on definitions and previous treatment strategies, and is thus a less precise definition of advanced stage of disease. A comparison between our cohorts and subsequent pooling of data is therefore justified as also supported by the multivariate analysis. Research in chemotherapy naive patients should be the focus of future studies.

Higher baseline levels showed a strong correlation with poor prognosis, first when analysed in the individual phase II studies, second when assessed according to quartiles of cfDNA levels in the total CRC cohort, and finally when divided into high and low concentration groups based on the maximum cfDNA level in healthy individuals. Furthermore, the combined analysis allowed for a reliable multivariate analysis, which confirmed a strong prognostic impact of cfDNA as a continuous parameter. The prognostic value of cfDNA has been described in various cancers, but only a few in relation to chemotherapy[15–16, 27]. The recently published report by Perkins et al is in agreement with our data and is one of the few studies investigating cfDNA in patients treated for advanced cancer[8].

Therapy for CRC will undoubtedly continue to develop towards targeting individual molecular characteristics of the disease. However, tumour heterogeneity, genomic instability, and clonal molecular evolution place high demands on a precise and timely characterisation. Performing repeated tumour biopsies has been done, but is inconvenient and involves a risk for the patient. Using plasma as a liquid biopsy for mutational detection has many advantages and could overcome the issues associated with repeated tumour biopsies. Repeated testing during therapy is easily done and the present method developed for detecting *KRAS* mutations is feasible, relatively cheap, fast and can be performed on a large-scale basis. However, the present qPCR technology will only allow a limited number of PCR reactions run per sample. Since *KRAS* has a predominant clinical impact and a high frequency in mCRC this approach is still feasible, whereas methods such as next generation sequencing may become relevant if multiple mutations with potential clinical importance and available targets are revealed. However, at this time, a simple and feasible method for these investigations seems the most rational approach, especially considering the fact that adequate sample sizes for reliable clinical investigations are of utmost importance.

The detection rate of tumour-specific mutations in plasma depends on the specificity and sensitivity of the assay, which can be increased by pre-analysis amplification steps, as well as on the amount of the cfDNA in the sample. The latter may be overcome by increasing the initial volume of plasma analysed. These aspects need to be considered when addressing the concordance between primary tumour and plasma mutation status, which was high with our method compared to the few relevant studies in the literature[28–30]. Reasons for discordance include the possible poor quality of the DNA extracted from paraffin embedded archival tumour tissue, or true disease heterogeneity and clonal molecular evolution during several lines of therapy.

It is clinically relevant to investigate if p*KRAS* can be used as a selection criterion for EGFR inhibitor treatment instead of t*KRAS* detection. The present data and a similar previous report[31] suggest that p*KRAS* status holds a strong predictive value in this setting. This also applies to the cohorts of our patients who were not treated with EGFR inhibitors. However, results from our small non-randomised studies should be interpreted with caution and further investigations in larger sample sizes, preferably in randomised studies, are warranted.

We have provided test and validation cohorts with adequate sample sizes for multivariate analysis of the prognostic value and for the definition of a normal range of cfDNA. The present study confirms the prognostic utility of cfDNA in plasma and feasibility for mutational testing with the method presented. We call for combined efforts to compare, validate and prospectively investigate the role of cfDNA quantification in cancer, with the overall perspective of translating results into clinical care of patients with advanced cancer diseases.
